# HLA imputation and its application to genetic and molecular fine-mapping of the MHC region in autoimmune diseases

**DOI:** 10.1007/s00281-021-00901-9

**Published:** 2021-11-16

**Authors:** Tatsuhiko Naito, Yukinori Okada

**Affiliations:** 1 Department of Statistical Genetics, Osaka University Graduate School of Medicine, 2-2 Yamadaoka, Osaka, Suita 565-0871 Japan; 2 Department of Neurology, Graduate School of Medicine, The University of Tokyo, Tokyo, Japan; 3 Laboratory of Statistical Immunology, Immunology Frontier Research Center (WPI-IFReC), Osaka University, Suita, Japan; 4 Integrated Frontier Research for Medical Science Division, Institute for Open and Transdisciplinary Research Initiatives, Osaka University, Suita, Japan

**Keywords:** HLA, HLA imputation, MHC, Fine-mapping, Autoimmune diseases

## Abstract

Variations of human leukocyte antigen (HLA) genes in the major histocompatibility complex region (MHC) significantly affect the risk of various diseases, especially autoimmune diseases. Fine-mapping of causal variants in this region was challenging due to the difficulty in sequencing and its inapplicability to large cohorts. Thus, HLA imputation, a method to infer HLA types from regional single nucleotide polymorphisms, has been developed and has successfully contributed to MHC fine-mapping of various diseases. Different HLA imputation methods have been developed, each with its own advantages, and recent methods have been improved in terms of accuracy and computational performance. Additionally, advances in HLA reference panels by next-generation sequencing technologies have enabled higher resolution and a more reliable imputation, allowing a finer-grained evaluation of the association between sequence variations and disease risk. Risk-associated variants in the MHC region would affect disease susceptibility through complicated mechanisms including alterations in peripheral responses and central thymic selection of T cells. The cooperation of reliable HLA imputation methods, informative fine-mapping, and experimental validation of the functional significance of MHC variations would be essential for further understanding of the role of the MHC in the immunopathology of autoimmune diseases.

## Introduction

The major histocompatibility complex (MHC) region is located at 6p21.3 with spanning approximately 5 Mb in length [1]. The genes encoded by this region are clearly enriched for immune responses and inflammatory pathways [1, 2]. Consistently with its function, genetic variants in the MHC region contribute to the genetics of various human complex traits, especially autoimmune diseases and infectious diseases [3, 4]. The MHC is the region with the highest number of disease associations reported in genome-wide association studies (GWAS) [5]. These associations included those “non-autoimmune diseases,” such as cardiovascular, metabolic, and neurological diseases, implying immune-related mechanisms behind the progression of these diseases and the broader significance of the MHC region [6, 7]. Among the genes densely present in the MHC region, human leukocyte antigen (HLA) genes are considered to explain most of the genetic heritability of MHC. HLA molecules mediate antigen presentation, which is a critical component in triggering the subsequent immune responses; thus, variations in HLA genes have been considered to associate with the risk of immune-related diseases directly. For a representative instance, in type 1 diabetes (T1D), the MHC region explains 42.8% of phenotypic variance, of which *HLA-DRB1*, *-DQA1*, and *-DQB1* haplotypes account for the most significant proportion at 29.6% [8].

Associations of single nucleotide polymorphisms (SNPs) with phenotypes of interest in GWAS typically do not indicate their direct causal roles but linkage with truly causal variants. To identify such causal variants (i.e., fine-mapping), comprehensive genotyping of regional variations including HLA allelic types for the target individuals is needed. However, the MHC region is one of the most challenging regions of the human genome to genotype because of its high degree of polymorphism and structural variations [9]. Thus, HLA typing is conducted with specific approaches, including traditional polymerase chain reaction (PCR)-based methods and next-generation sequencing (NGS). They are so labor-intensive, time-consuming, and expensive that they could not be applied to fine-mapping for large cohorts of GWAS [6, 10]. Subsequently, the genotypes of HLA alleles are indirectly imputed from SNP-level data using a pre-constructed HLA reference panel. HLA imputation has successfully contributed to the fine-mapping of causal HLA variants to delineate of the immunopathology of various diseases.

Beginning with a simple inference using tag SNPs [11, 12], various statistical HLA allelic imputation methods have been developed, each with its advantages and disadvantages for practical use. In this review, we discuss the recent advances and challenges in HLA imputation methods and available HLA reference panels. We also discuss the relationship between the MHC region and autoimmune diseases revealed by the fine-mapping and the current understanding of how HLA variations contribute to disease etiology.

## Structure and definition of HLA

**Fig. 1:**
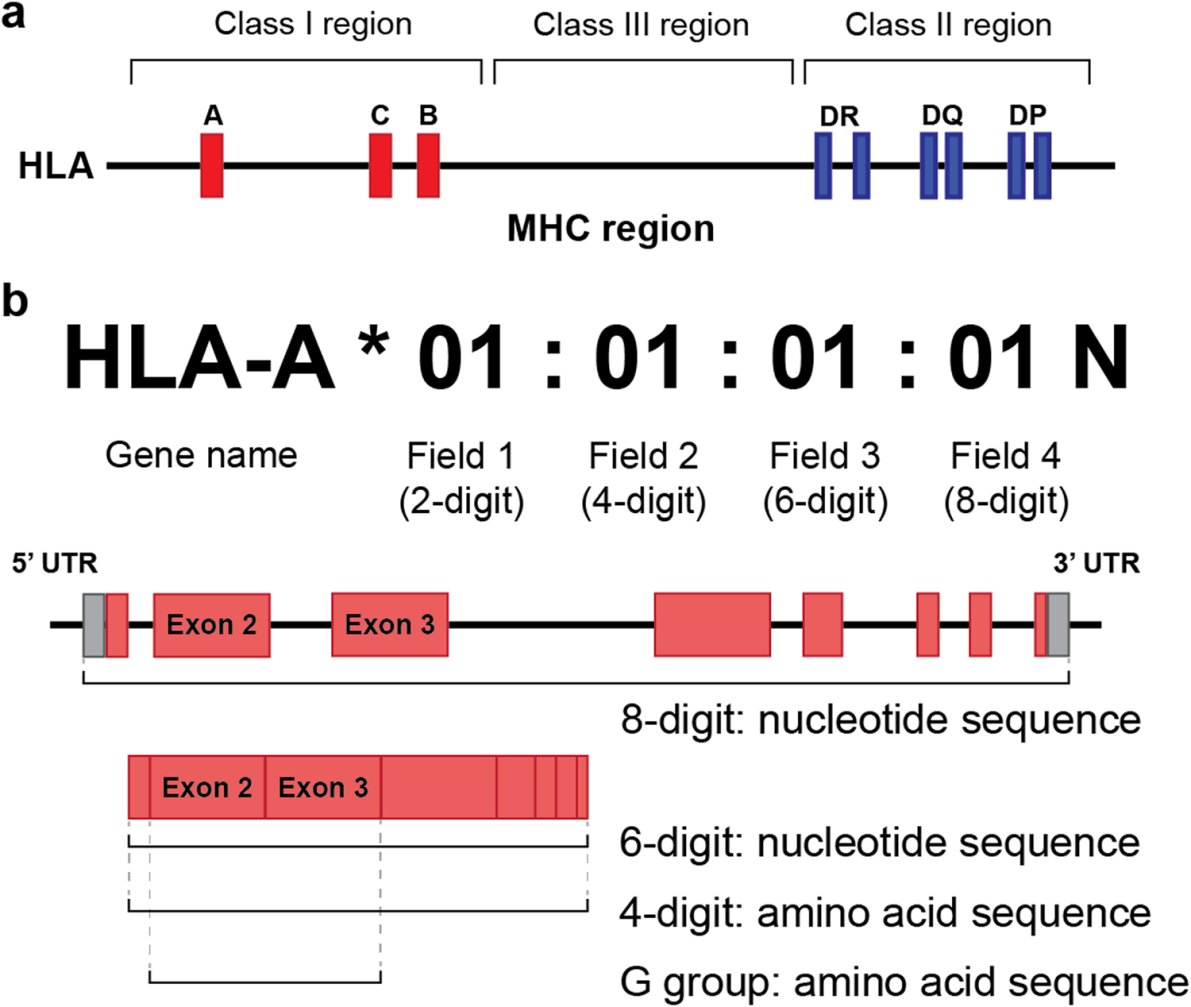
Structure of the MHC region and nomenclature of HLA alleles. **a** The MHC region is categorized into class I, II, and III. Only classical HLA genes are illustrated along with their positions for simplicity. **b** The nomenclature of HLA alleles. HLA alleles are named hierarchically as four fields based on the resolution of sequences. The last letter denotes expression status, e.g., “N” indicates “not to be expressed.”

The MHC region is categorized into three sub-regions, namely, class I, II, and III (Fig. 1a) [1]. In the MHC class I region, three categories of genes are located: classical HLA class I genes (*HLA-A*, *-B*, and *-C*), non-classical HLA class I genes (*HLA-E*, *-F*, *-G*, *HFE*, and 12 pseudogenes), and the class I-like genes (*MICA*, *MICB*, and 5 pseudogenes). In the MHC class II region, there are two categories of HLA genes: classical HLA class II genes (*HLA-DR*, *-DP*, and *-DQ*) and non-classical HLA class II genes (*HLA-DM* and *-DO*). The remaining part is the class III region, where many of the genes are related to the immune system, such as complement (e.g., *C2*, *C4A*, and *C4B*) and inflammation system (e.g., *TNF*).

HLA class I molecules are expressed on the surface of nucleated cells and can present endogenous antigens to CD8+ T cells. While classical HLA class I genes are highly polymorphic and have distinct antigen-presenting ability, non-classical HLA class I genes are less polymorphic and have various functions. The structure of HLA class I molecules consists of a heavy chain consisting of three domains, α1, α2, and α3, and β2 microglobulin that constitutes one immunoglobulin-like domain.

HLA class II molecules are expressed on the surface of antigen-presenting cells, such as macrophages and dendritic cells, and function to present exogenous antigens to CD4+ T cells. The structure of HLA class II molecules consists of an alpha chain composed of two domains, α-domain consisting of α1 and α2 and β chain consisting of β1 and β2. Each HLA class I molecule (e.g., A, B, and C) is encoded by a single gene (e.g., *HLA-A*, *-B*, and *-C*, respectively). In contrast, for HLA class II, the heterodimer is formed from the products of two genes, e.g., *HLA-DQA1* and *HLA-DQB1* encode the α and β chains of DQ molecules, respectively. Although β chains of DR molecules are encoded by *HLA-DRB1*, there are additional loci encoding alternative DRβ chains in some haplotypes (e.g., *HLA-DRB3*, *-DRB4*, and *-DRB5*). The presence of the additional loci depends on the serogroup of *HLA-DRB1* gene on the same haplotype and named accordingly (e.g., *HLA-DRB4* corresponds to HLA-DRB1*04).

The rapid increase in the number of identified HLA alleles has led to the development of the nomenclature used to describe them. It is managed by the WHO Nomenclature Committee for Factors of the HLA System, and all identified HLA alleles are registered in the IMGT/HLA database [13]. In the HLA allele nomenclature, the HLA gene name is followed by numeric fields separated by colons that describe four levels of typing resolution (Fig. 1b). The first field or 2-digit resolution describes a serologically defined allele group, and the second field or 4-digit resolution indicates a unique protein sequence encoded by the allele within that group. Fields 3 and 4 resolutions show silent and non-coding polymorphisms, respectively. Traditionally, HLA typing was mainly based on the antigen-binding region (i.e., exons 2 and 3 for an HLA class I gene and by exon 2 for an HLA class II gene). However, with the increase in the number of HLA types registered in the database, many polymorphisms have been found outside the antigen-binding region. As a result, the G group was defined as a type of group in which the sequence of the antigen-binding region (i.e., exons 2 and 3 for class I and exon 2 for class II genes) to differentiate it from the 4-digit resolution allele [14].

## HLA imputation methods for individual genotype data

**Fig. 2:**
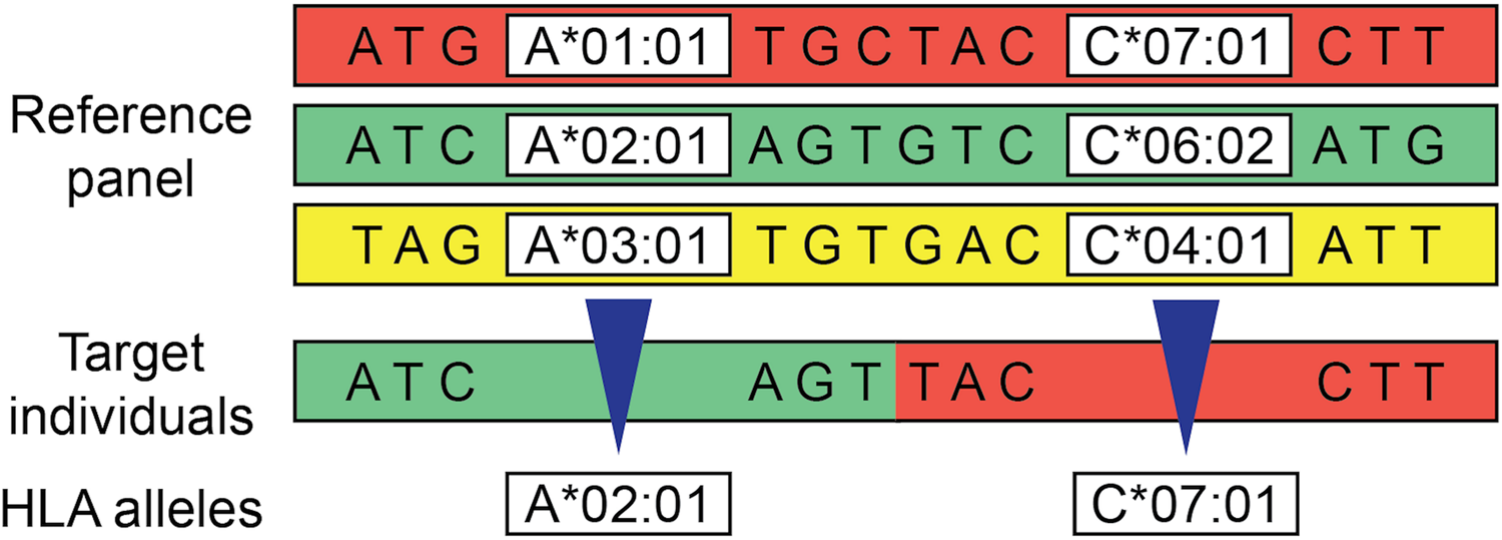
An illustration of HLA imputation using a reference panel. An HLA reference panel contains individual data for which both SNP genotypes and HLA typing information are available. Based on LD information from a reference panel, it is possible to infer HLA allelic information of target individuals for whom only SNP genotype information is available.

HLA imputation was developed to fine-map the MHC region, characterized by complicated linkage disequilibrium (LD) structures and long-range haplotypes of regional variants and their corresponding HLA allelic types. HLA imputation uses a reference panel typed with both HLA and SNP genotypes to infer HLA genotypes from SNP information (Fig. 2). Starting with a simple inference using tag SNPs [11, 12], various HLA imputation methods have been developed to capture the complicated LD structure of the MHC region (Table 1).

**Table 1:** Comparison of HLA imputation software

Name	Type	Methods	URL	Reference
HLA*IMP:02	Stand-alone software	Haplotype-graph model	NA	[15]
HLA*IMP:03	Web application	Random forest model	http://imp.science.unimelb.edu.au/hla/	[16]
SNP2HLA	Shell script	Beagle with considering markers as binary alleles	http://software.broadinstitute.org/mpg/snp2hla/	[17]
HIBAG	R package	Bagging of multiple classifiers of EM algorithm	https://github.com/zhengxwen/HIBAG	[18]
CookHLA	Python script	Beagle with considering markers as binary alleles and embedding of markers on exons	https://github.com/WansonChoi/CookHLA	[19]
DEEP*HLA	Python script	Multi-task convolutional deep neural networks	https://github.com/tatsuhikonaito/DEEP-HLA	[20]

Leslie et al. first reported a probabilistic approach for classical HLA allelic imputation based on the Li and Stephens haplotype model [21]. Its improved version was implemented as HLA*IMP targeted for the European population [22]. The Li and Stephens haplotype model is a theory of statistical genetics, stating that the genome sequence of an individual can be represented by recombination and a small number of mutations of those of other individuals [23]. They modeled the SNP haplotype background of individual HLA alleles and performed Bayesian inference to determine genotypes of HLA alleles [23]. Dilthey et al. developed a subsequent software program, HLA*IMP:02, which uses a haplotype graph approach with SNP data from multiple populations to address haplotypic heterogeneity [15]. HLA*IMP:02 is currently available in Thermo Fisher Scientific software for samples typed with its SNP-genotyping array. HLA*IMP:03 is web-based software, which uses random forest models [16]. SNP2HLA adopts an innovative approach in which multi-alleles of HLA genes are viewed as individual binary alleles and are imputed using Beagle, standard SNP genotype imputation software based on a haplotype graph approach [17]. One of its advantages is that SNP2HLA imputes HLA types and amino acid allele genotypes simultaneously. HIBAG (HLA Genotype Imputation with Attribute Bagging) estimates the likelihood of HLA alleles by the ensemble of multiple classifiers that model haplotypes and their frequencies based on expectation-maximization algorithm [18].

For widely used software, overall accuracy for high-quality reference panels is greater than 90% [24]. However, their accuracy tends to significantly decline as alleles were less frequent [19, 20]. Additionally, imputation accuracy in hyper-multi-allelic genes, such as *HLA-B* and *HLA-DRB1*, drops. In contrast, recently developed techniques presented their improvement of accuracy in such respects. CookHLA is similar to SNP2HLA in that it treats the multi-allelic HLA information as a set of binary markers but has several updates [19]. While SNP2HLA places each marker set in the center position of the gene, CookHLA embeds each marker set in the middle position of each polymorphic exon (i.e., exons 2, 3, and 4 for class I genes; and exons 2 and 3 for class II genes). It addresses the issue of LD decay with distance by effectively capturing the information of polymorphic exons. CookHLA repeats imputation for each exon and combines the posterior probabilities to make final consensus calls. Furthermore, CookHA uses Beagle v4 instead of v3, which was built in SNP2HLA. CookHLA achieved higher accuracy than SNP2HLA and HIBAG with significant superiority for less frequent alleles. For instance, CookHLA achieved 80% accuracy in alleles in frequency 0.1–0.5% for a European reference panel, whereas conventional methods presented 40−60% accuracy.

**Fig. 3:**
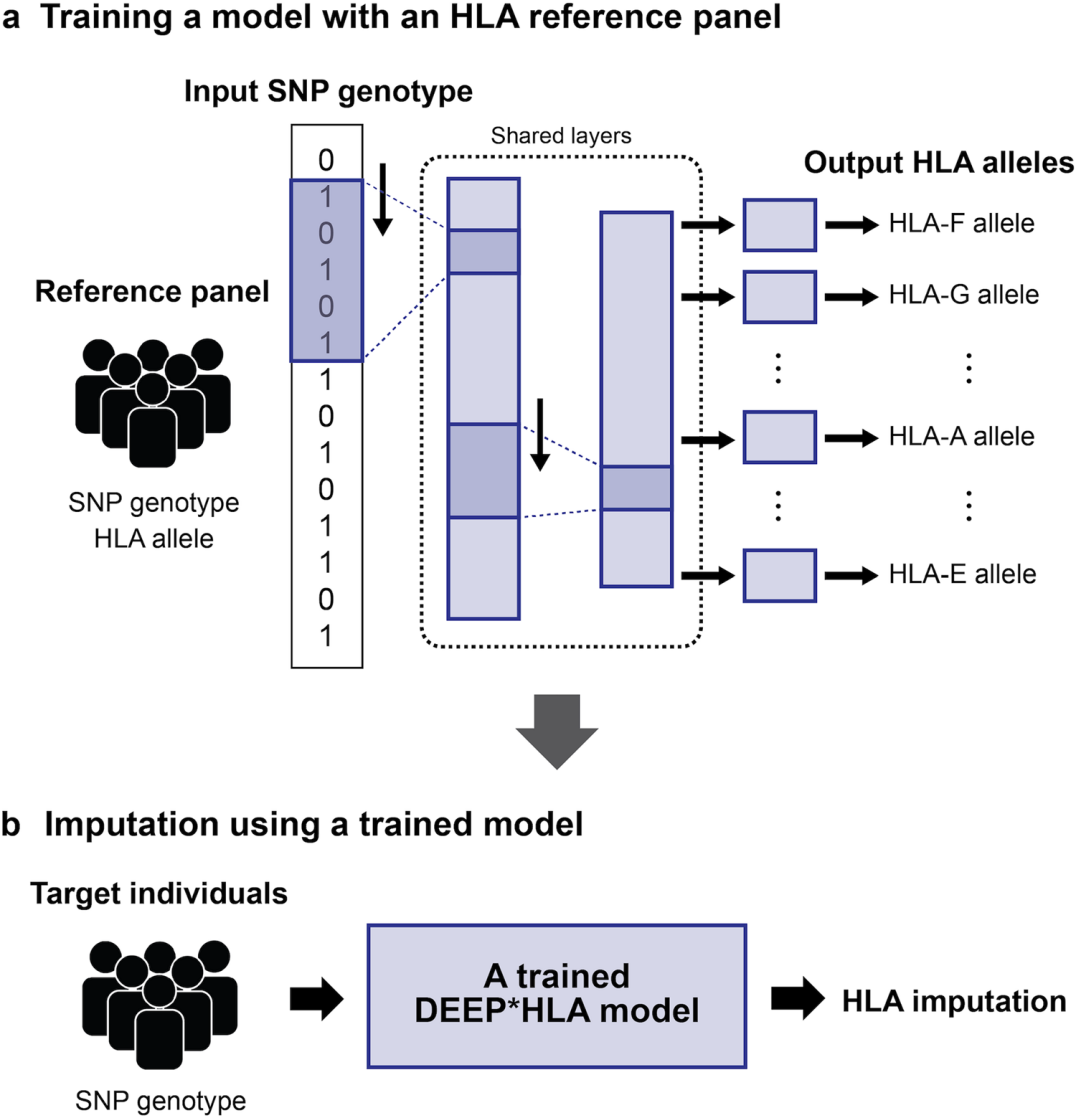
The architecture and imputation strategy of DEEP*HLA. DEEP*HLA is a multi-task deep convolutional neural network model that takes SNP information and outputs genotype probabilities of HLA genes. In the training phase, DEEP*HLA learns the relationship between SNP genotype and HLA alleles from an HLA reference panel consisting of many individuals (**a**). In the imputation phase, a trained DEEP*HLA can perform HLA imputation from SNP genotype data without a reference panel (**b**).

DEEP*HLA is also a recently published software, which uses a deep learning model to capture the complex LD structure of the MHC region. It utilizes the advantage of multi-task convolutional neural networks [20], which takes SNP input and impute alleles of multiple HLA genes belonging to the same preset group simultaneously (Fig. 3). Conventional imputation algorithms based on the Markov model of sequential information would show limited performance for imputing alleles without distant-dependent LD decay features. In contrast, DEEP*HLA was less dependent on distant-dependent LD decay, thanks to the nature of neural networks. DEEP*HLA was advantageous, especially for its low-frequency and rare alleles. It achieved around 80% accuracy for alleles with a frequency < 1% in most settings, while conventional methods presented 60−70% accuracy. Furthermore, DEEP*HLA was computationally efficient enough to be applied to biobank-scale data.

One aspect that determines which imputation software should be used is whether you have an HLA reference panel for a target population. HLA*IMP:02 and HLA*IMP:03 are pretrained with their reference data; thus, there is no need for your own. In contrast, the current version of HLA*IMP:02 and HLA*IMP:03 does not support a function for users to generate an imputation model using their own data locally. While SNP2HLA and CookHLA explicitly use reference haplotype data always, HIBAG and DEEP*HLA do not require these data once the trained models are generated. Since it is difficult to restore genotype information of individuals from the model parameters, their trained models could be publicly distributed or moved without ethical permission.

The formats of genotype data and HLA reference and nomenclature of HLA alleles are often unorganized, so that HLA association analysis has been laborious. HLA-TAPAS (HLA-Typing At Protein for Association Studies) is a sophisticated integrated pipeline, including data formatting, HLA reference panel construction, HLA imputation, and HLA association analysis. The imputation method of HLA-TAPAS adopts that of SNP2HLA in which Beagle v4 is used, unlike the original SNP2HLA software [25, 26]. As is the case with CookHLA, Beagle v4 supports multithreading, which would make it applicable for biobank-scale data.

## HLA imputation for GWAS summary statistics

While privacy and ethical constraints often restrict access to individual GWAS genotype data, sharing GWAS summary statistics has become more prevalent. Imputation of summary statistics has been developed in this context [27]. In summary, statistics-based imputation and associations of untyped alleles are inferred from the approximation of Z scores to a multivariate normal distribution. Li et al. extended this algorithm to the imputation of HLA association tests as DISH (Direct Imputing Summary association statistics of HLA variants) software [28]. Although it might be challenging to perform a detailed fine-mapping, such as haplotype analysis, reliable conditional analysis, and additional adjustment of covariates, DISH would play a sufficient role in obtaining meaningful inferences. We recently conducted trans-ethnic fine-mapping for Parkinson’s disease (PD) by integrating GWAS summary statistics from different studies to detect functionally plausible risk-associated HLA variants [7].

## Existing HLA imputation reference panels and challenges

A high-quality HLA reference panel is a prerequisite to achieve high-performance of HLA imputation. We summarized available HLA imputation reference panels in Table 2. In traditional HLA reference panels, HLA typing was conducted with PCR-based genotyping methods [17, 32, 34]. PCR-based HLA typing methods are limited to G group resolution or frequent alleles.

**Table 2:** A list of existing HLA reference panels

Population	HLA typing method	Sample size	URL	Year	Reference
Europeans	SSOP	5225	http://software.broadinstitute.org/mpg/snp2hla/	2013	[29]
Korean	NGS	413	https://sites.google.com/site/scbaehanyang/hla_panel	2014	[30]
East and South Asians	SSOP	530	http://software.broadinstitute.org/mpg/snp2hla/	2014	[31]
Japanese	SSOP	908	https://humandbs.biosciencedbc.jp/hum0028-v2	2015	[32]
Han-Chinese	NGS	10,689	http://gigadb.org/dataset/100156	2016	[33]
Japanese	NGS	1120	https://humandbs.biosciencedbc.jp/en/hum0114-v2	2019	[6]
Finnish	SSOP, SSP, SBT	1150	NA	2020	[34]
Europeans	NGS	401	NA	2020	[35]
Taiwanese	NGS	1012	NA	2020	[36]
Multi-ethnicity	NGS	21,546	https://github.com/immunogenomics/HLA-TAPAS/	2021	[26]

*SSOP* sequence-specific oligonucleotide probe, *NGS* next-generation sequencing, *SSP* sequence-specific primer, *SBT* sequencing-based typing

In contrast, high-throughput of NGS technologies and sophisticated HLA type inference algorithms has enabled higher resolution typing using either exons only or larger gene segments, including whole HLA genes [37, 38]. Reliance on a single reference sequence would be problematic in the assembly since MHC haplotypes have significant variations in genomic contents and length [1]. Thus, as exemplified by the genome graph-based methods developed by Dilthey et al. [39, 40], HLA type inference methods focusing on sequence diversity have been developed [41, 42]. Explanation of HLA type inference methods would be beyond the scope of this review and has been well discussed elsewhere [43]. Recently published HLA reference panels have mainly used NGS technologies. Kim et al. constructed a Korean reference panel with a hybrid method of the SSO method for *HLA-DRB1* and NGS for other HLA genes [30]. Zhou et al. performed deep sequencing of the MHC region and constructed a Han-Chinese reference panel containing 10,689 normal healthy controls [33]. Hirata et al. constructed a Japanese reference panel based on NGS, which covered high-resolution allelic information of the extended MHC region, including non-classical HLA genes [6]. Squire et al. also constructed a reference panel mainly of Europeans, which included non-classical HLA and HLA-like genes [35].

Conventional HLA reference panels have been constructed for a single ancestry, considering that the LD and haplotype structure of the MHC region is highly ancestry specific. Thus, the ancestral background of a reference panel must be as close as possible to a target population. In contrast, a multiethnic reference panel is constructed with the expectation that its diversity could cover ethnically heterogeneous populations. Degenhardt et al. constructed a multiethnic reference panel by integrating multiple existing single-ancestry reference panels and demonstrated that it could maintain high accuracy across different ethnicities [29]. Luo et al. constructed a large-scale multiethnic reference panel (*n* = 21,456), including Admixed African, East Asian, European, and Latino, with short-read whole sequencing data. It achieved an accuracy of >90% at the G group resolution in all the ancestries. This reference panel is also publicly available in the Michigan Imputation Server, allowing direct imputation using this panel [44].

The currently available reference panels based on short-read NGS technologies could not capture the entire MHC region in a haplotype-preserving manner. Assembly of complete MHC haplotypes has been challenging due to several reasons, such as sequence homologies between the HLA genes and large structural variants, especially in the MHC class II region and class III region (e.g., C4 genes). This is problematic since such regions with high levels of structural variations may have a greater effect on the risk of some diseases. Indeed, it has been shown that structural variants in the C4 genes can largely explain the MHC genetic risk of schizophrenia [45]. Furthermore, Kamitaki et al. revealed that the C4 allele associated with the risk of schizophrenia could have a protective effect on SLE and SjS by imputation of C4 structural haplotypes using WGS data [46]. They demonstrated that the C4 gene variant explains the risk of SLE rather than its tagged HLA-DRB1*03:01, which had been presumed as the risk itself. In these diseases, the MHC genetic predisposition might be attributed not to precise interactions to specific autoantigens but to the continuous interaction of the immune system with a large number of potential autoantigens, which are modulated by C4 protein. These observations imply that fine-mapping using the current reference panels might distort our interpretation of the role of HLA variants in the etiology of some diseases and thus need to be improved.

In contrast, long-read sequencing technologies have been attracting attention as a potential method to solve these problems. Koren et al. reported the trio binning-based assembly of a diploid MHC with perfect HLA typing results [47]. Equivalent results were obtained with nanopore ultra-long reads [48]. Based on a combination of state-of-the-art long and short reads, Chin et al. produced a high-quality diploid MHC assembly of HG002, one of the GIAB benchmarking samples [49]. Future reference panels will take advantage of these technologies to realize more thorough MHC fine-mapping. Considering the current cost of the long-read sequencing technologies, the next step might be the flow of information from a relatively small number of long-read-sequenced samples to the refinement of HLA reference panels constructed by short-read sequencing, which could be used for HLA imputation from SNP array data [43].

## Findings obtained from fine-mapping of the MHC region

In 2010, the association of HIV controllers was first mapped to specific amino acids of HLA class I genes through HLA imputation [50]. In 2012, as one of the first representative studies of MHC fine-mapping on an autoimmune disease using HLA imputation, the risk of rheumatoid arthritis (RA) in European populations was mapped to independent associations of amino acid alleles in HLA class I and II genes [51]. While the risk-HLA alleles had been conventionally reported for a set of amino acid positions 70–74 in HLA-DRβ1 (i.e., “shared epitope”), the study showed that the strongest association was fine-mapped to amino acid position 11 in HLA-DRβ1.

Since then, HLA imputation has successfully contributed to the fine-mapping in the MHC region on various autoimmune diseases. The risk amino acid positions of RA were replicated in East-Asian populations [31, 52], and additionally, the risk contributions of *HLA-DOA* were also identified [53]. The MHC risk of other autoimmune diseases was also fine-mapped, including systemic lupus erythematous (SLE) [52, 54], dermatomyositis [55], idiopathic inflammatory myositis [56], juvenile idiopathic arthritis [57], Sjögren’s syndrome (SjS) [58], polyangiitis [59, 60], ankylosing spondylitis (AS) [61], psoriasis [33, 62, 63], celiac disease [64], T1D [8, 65], Graves’ disease [32], inflammatory bowel diseases [66, 67], pulmonary alveolar proteinosis (PAP) [68], primary biliary cholangitis [69–71], and multiple sclerosis [72, 73]. Attempts to find novel insights by inter-ethnic comparison or integration by trans-ethnic fine-mapping have also been made for T1D [20] and ulcerative colitis [74].

For most common autoimmune diseases, the major risk-associated HLA loci were known from epidemiological studies; thus, fine-mapping studies have contributed to the confirmation of such associations at the level of specific variants and the additional identification of independent associations in different HLA loci. For instance, a fine-mapping study of AS revealed that not only the well-known HLA-B*27 alleles but also different *HLA-B* alleles were associated with the risk [61]. Interestingly, the stratified analysis demonstrated that a variant in *ERAP1*, which encodes a protein involved in peptide trimming in HLA class I presentation, was correlated with the risk in carriers of specific *HLA-B* alleles. In addition to *HLA-B*, variants in *HLA-A*, *-DPB1*, and *-DRB1* were independently associated with the risk of AS. In some diseases, the association with HLA itself has been proven by fine-mapping. PAP is a rare disease, in which autoimmunity to pulmonary surfactant contributed to the pathogenesis. An MHC find-mapping study first revealed that a specific *HLA-DRB1* allele confers its major genetic risk [68]. We summarized the current findings on risk-associated HLA loci and independent associations of specific HLA variants for autoimmune diseases obtained from MHC fine-mapping studies in Table 3. We note that these findings may be updated through refinement of reference panels as mentioned in the example of C4 [46] or functional fine-mapping as described later.

**Table 3:** Major findings on HLA associations in autoimmune diseases obtained from MHC fine-mapping studies

Disease	HLA class (top association)	HLA loci associated with risk	Independently associated HLA variants	Population	Reference
Rheumatoid arthritis	II	*HLA-DRB1*, *HLA-B*, *HLA-DPB1*	HLA-DRβ1 AA 11 and 13, 71, 74; HLA-B AA 9; HLA-DPβ1 AA 9	European	[51]
	II	*HLA-DRB1*, *HLA-B*, *HLA-DPB1*	HLA-DRβ1 AA 11 and 13, 57, 74; HLA-B AA 9; HLA-DPβ1 AA 9	East Asian	[31]
	II	*HLA-DRB1*	HLA-DRβ1 AA 11 and 13	East Asian	[52]
	II	*HLA-DRB1*, *HLA-DPB1*, *HLA-DOA*, *HLA-B*	HLA-DPβ1 AA 84; rs378352 (HLA-DOA); HLA-B*40:02	East Asian	[53]
Systemic lupus erythematous	II	*HLA-DRB1*	HLA-DRβ1 AA 11 and 13	East Asian	[52]
	II	*HLA-C*, *HLA-B*, *HLA-DRB1*, *HLA-DQA1*, *HLA-DQB1*	HLA-DRB1∗15:03; HLA-DQB1∗02:02, 03:19; HLA-DQA1∗05:01 02:01, 05:05; HLA-B∗08:01; HLA-C∗17:01	African	[54]
	II	*HLA-DQB1*, *HLA-B*, *HLA-DRB3*, *HLA-DQA1*	HLA-DQB1∗02:01; HLA-B∗08:01, 18:01; HLA-DRB3∗02; HLA-DQA1∗01:02	European	[54]
Dermatomyositis	II	*HLA-DPB1*	HLA-DPB1*17	East Asian	[55]
Idiopathic inflammatory myositis	II	*HLA-DRB1*, *HLA-B*, *HLA-DQB1*	HLA-DRB1*03:01; HLA-B*08:01; HLA-DQβ1 AA 57; HLA-DQB1*04:02	European	[56]
Juvenile idiopathic arthritis	II	*HLA-DRB1*, *HLA-DPB1*, *HLA-A*, *HLA-B*	HLA-DRβ1 AA 13; HLA-DPB1*02:01; HLA-A AA 95; HLA-B AA 152	European	[57]
Sjögren's syndrome	II	*HLA-DQB1*	HLA-DQB1*0201	European	[58]
Granulomatosis with polyangiitis	II	*HLA-DPB1*	HLA-DPB1*04	European	[59]
Eosinophilic granulomatosis with polyangiitis	II	*HLA-DRB1*, *HLA-DQA1*, *HLA-DRB1*	HLA-DRB1*08:01; HLA-DQA1*02:01; HLA-DRB1*01:03	European	[60]
Ankylosing spondylitis	I	*HLA-B*, *HLA-A*, *HLA-DPB1*, *HLA-DRB1*	HLA-B*27, 07:02 and 57:01; HLA-A*02:01; rs1126513 (HLA-DPB1); HLA-DRB1*01:03	European	[61]
Psoriasis	I	*HLA-C*, *HLA-B*, HLA-DPB1	HLA-C*06:02, 07:04; HLA-B AA 9, 67; HLA-DPB1*05:01	East Asian	[33]
	I	*HLA-A*, *HLA-C*, *HLA-DQB1*	HLA-A*02:07; HLA-C*06:02; HLA-DQβ1 AA 57	East Asian	[62]
Celiac disease	II	*HLA-DQB1*, *HLA-DQA1*	HLA-DQβ1 AA 74, 57; HLA-DQα1 AA 47, 25	European	[63]
Type I diabetes	II	*HLA-DQB1*, *HLA-DRB1*, *HLA-B*, *HLA-A*	HLA-DQβ1 AA 57, HLA-DRβ1 AA 13, 71; HLA-B*39:06; HLA-DPB1*04:02; HLA-A AA 62	European	[8]
	II	*HLA-DQB1*, *HLA-DRB1*, *HLA-A*, *HLA-C*	rs1770 (HLA-DQB1); HLA-DRβ1 AA 74, 11; HLA-A AA 9; HLA-C AA 275	East Asian	[65]
	II	*HLA-DQB1*, *HLA-DRB1*, *HLA-B*, *HLA-A*	HLA-DQβ1 AA 185, 30, 70; HLA-DRβ1 AA 71, 74; HLA-B*54:01; HLA-A AA 62	East Asian, European	[20]
Grave's disease	II	*HLA-DPB1*, *HLA-A*, *HLA-B*, *HLA-DRB1*	HLA-DPβ1 AA 35, 9; HLA-A AA 9; HLA-B AA 45, 67; HLA-DRβ1 AA 74	East Asian	[32]
Crohn's disease	II	*HLA-DRB1*, *HLA-C*	HLA-DRB1*01:03; HLA-C*06:02	European	[66]
	II	*HLA-DRB1*, *HLA-DQB1*	HLA-DRβ1 AA 37, 57, HLA-DRB1*04:03	East Asian	[67]
Ulcerative colitis	II	*HLA-DQA1*, *HLA-DRB1*, *HLA-C*	rs6927022(HLA-DQA1); HLADRB1*01:03; HLA-C*12:02	European	[66]
Pulmonary alveolar proteinosis	II	*HLA-DRB1*	HLA-DRB1*08:03; HLA-DPβ1 AA 8	East Asian	[68]
Primary biliary cholangitis	II	*HLA-DRB1*, *HLA-DPB1*	HLA-DRB1*08 and 14; HLA-DPB1*03:01	European	[69]
	II	*HLA-DPB1*, *HLA-DRB1*	HLA-DPβ1 AA 11; HLA-DRβ1 AA 74; HLA-DQβ1 AA 57; HLA-C AA 155; HLA-DQα1 AA 13	European	[70]
	II	*HLA-DRB1*, *HLA-DQB1*, *HLA-DPB1*	HLA-DRβ1 AA 74; HLA-DQβ1 AA 55; HLA-DPβ1 AA 85, 55	East Asian	[71]
Multiple sclerosis	II	*HLA-DRB1*, *HLA-A*, *HLA-DPB1*, *HLA-B*	HLA-DRB1*15:01, 03:01, 13:03, 04:04, 04:01, 14:01; HLA-A*02:01; HLA-DPβ1 AA 65; rs2516489; HLA-B*37:01, 38:01	European	[72]
	II	*HLA-DRB1*, *HLA-A*, *HLA-DPB1*, *HLA-B*, *HLA-DQA1*, *HLA-DQB1*	HLA-DRB1*15:01, *03:01, *13:03, *08:01; HLA-A*02:01, rs9277565 (HLA-DPB1); HLA-B*44:02, *38:01, *55:01; rs2229029 (LTA)	European	[73]

*AA* amino acid

Not confined to so-called autoimmune diseases, MHC fine-mapping studies have successfully identified the risk HLA variants of different diseases, such as infectious diseases [75, 76], malignant tumors [77, 78], and neurological diseases [7, 79]. These studies could expand our knowledge of the involvement of autoimmunity in the progression of such diseases. For instance, in PD, a neurodegenerative disease characterized by the deposition of protein aggregates containing α-synuclein, GWAS, and fine-mapping studies have suggested an association of the HLA variants with the risk [80, 81]. Subsequently, an aberrant T cell response to α-synuclein associated with *HLA-DRB1* alleles was revealed in PD patients, significantly advancing the understanding of the role of acquired immunity in the pathogenesis of PD [82]. Furthermore, phenome-wide fine-mapping of the MHC region revealed a wide variety of associations and the relationships among different phenotypes [6, 76, 83]. ﻿

## Current procedure and challenges in fine-mapping

A typical procedure of fine-mapping of the MHC region enables both exploration of independent HLA loci and disentanglement of independently associated variants in the locus. For instance, in the MHC fine-mapping on RA [51], the strongest associations were mapped to the *HLA-DRB1* region, followed by the *HLA-B* and *HLA-DPB1* regions by step-wise conditional analysis, wherein the locus with the strongest association are successively conditioned on. Then, independently associated variant sets consisting of amino acid or HLA alleles were detected by step-wise conditional analysis in individual loci. Independent effects of single variants are evaluated in an additive model, in which the effects of the two alleles on a disease of interest are independent and combine linearly.

In contrast, non-additive effects statistically mean deviation from this linear relationship, which may arise from interactions between two alleles or individual alleles’ inherent effects [84]. In terms of the function of HLA, an individual’s two expressed HLA alleles with different antigen-binding repertoires are speculated to present a synergic effect on antigen-presentation ability, leading to an extraordinary disease risk. MHC fine-mapping analysis has also been used to elucidate non-additive and interaction effects. Lenz et al. comprehensively evaluated the non-additive and interaction effects of several autoimmune diseases. They reported non-additive effects explained by interactions between specific HLA alleles in RA, T1D, and celiac disease [84]. Hu et al. reported that several combinations of haplotypes of *HLA-DRB1*, *-DQA1*, and *-DQB1* presented an association with the risk on T1D beyond an additive effect [8]. Interaction effects between MHC and non-MHC genes have also been reported in several autoimmune diseases, such as interaction with cytotoxic T lymphocyte antigen 4 (*CTLA4*) [85], several killer immunoglobulin receptor (KIR) genes [86, 87], and *ERAP1* and *2* [88, 89]. Variations in the KIR region can be imputed by a method similar to HLA imputation [90]. Thus, hybrid fine-mapping in the MHC region and KIR region would further our understanding on how they interactively associate with the risk of autoimmune diseases.

While focusing on the nominal statistical significance of all the variants in the MHC region is currently a standard approach, weighting or filtering of variants based on their functional annotations is an effective approach for fine-mapping in normal genomic regions [91]. Some variants have an eQTL effect on HLA genes [92, 93]. Furthermore, differential allelic expression of HLA genes has been reported in association with the etiology of several diseases [94, 95]. Considering these observations, functional fine-mapping would be helpful in further understanding the role of variations in the MHC region for disease etiology. Due to the difficulty in mapping short-reads of the highly polymorphic MHC loci and quantification of HLA gene expressions [96, 97], eQTL database is lacking, especially for different populations. The construction of an eQTL database for HLA genes based on a state-of-the-art NGS technology and mapping strategy would be expected [96–98].

## Functional contribution of the HLA risk allele

MHC fine-mapping studies have shown that the amino acid alleles composing HLA alleles are likely to have stronger associations than the classical HLA alleles [8, 51]. Typically, risk-associated amino acid polymorphisms of autoimmune diseases are located in peptide-binding grooves of HLA molecules, which are considered to lead to the altered binding affinity of HLA molecules to the autoantigen peptides [32]. The altered binding affinity by the causal HLA alleles can be experimentally validated using HLA-peptide binding assay [99, 100]. Otherwise, if the epitope of an autoantigen for a disease of interest is already known, in silico prediction tools for HLA binding affinity could also be helpful to obtain a meaningful inference [7, 101]. Antigen peptides presented by HLA molecules are recognized by T cell receptors (TCRs), leading to antigen-specific immune responses. Then, the altered interaction between HLA, peptides, and TCRs by variations in HLA alleles is presumed to influence the immune response by two major mechanisms: thymic selection of T cells and peripheral T cell response [75, 102].

In the mechanism associated with thymic selection, specific MHC/peptide–TCR interactions, which MHC variants could alter, will determine the selection of the T cell repertoire during primary tolerance events, leading to differential susceptibility to disease progression. The antigen specificity of the TCR is determined by hyper-variable complementary determining region 3 (CDR3) [103]. During T cell development in the thymus, a highly diverse CDR3 repertoire is generated through random VDJ recombination in immature T cells. In the positive selection, thymic T cells that bind moderately to MHC complexes would survive. Conversely, T cells whose TCRs bind too strongly to MHC complexes, which are likely to be self-reactive, are killed as the process of negative selection. For instance, T1D risk-associated DQ molecules present weak binding to an epitope and are likely to escape from negative selection [104, 105]. Not limited to binding affinity, thymic escape due to the protein instability of DQ molecules is also suggested to associate with the risk of T1D [106, 107]. Recently, Ishigaki et al. investigated the association between HLA allelic variations and CDR3 amino acid features through CDR3 quantitative analysis (cdr3-QTL) [102]. In this study, the HLA amino acid position that explained the most variance in CDR3 composition was position 13 in HLA-DRβ1, which is the strongest association to RA risk. The effect sizes of multiple amino acids in this position were consistent between the risk of RA and cdr3-QTL, which supported the assumption that HLA risk for RA is mediated by TCR composition in some degree. Furthermore, they integrated the risk for several autoimmune diseases throughout the MHC region as an HLA risk score and identified multiple CDR patterns associated with the risk of the diseases. Considering the overlap between cdr3-QTL and risk-associated HLA variants in autoimmune diseases, the cdr3-QTL information might be utilized as an annotation for functional fine-mapping in the MHC region.

The MHC/peptide–TCR interaction in peripheral T cell immune responses would also be influenced by altered binding affinity dependent on MHC variants and associated with disease susceptibility. For instance, citrullinated self-peptides tend to bind to RA risk-associated HLA-DR molecules stronger than non-RA risk-associated HLA-DR molecules [99]. It is unclear whether T-cell selection in the thymus or the peripheral T-cell response is the primary contributor to the pathogenesis and how they are related to each other. An interesting example regarding their relationship is the neoantigen hypothesis of the association between RA and the risk-HLA alleles [108]. The conversion of electrically positive arginine to electrically neutral citrulline at the P4 position of peptides, which interacts with the SE, significantly increases the binding affinity of SE-containing HLA-DR molecules [99]. This finding might suggest that SE-containing HLA-DR molecules fail to induce tolerance in thymic selection under non-inflammatory conditions because they cannot bind and present peptides with arginine residues at the P4 position. Then, P4-citrullinated self-peptides can be presented by SE-containing HLA-DR molecules and induce peripheral T cell response.

## Conclusions

We have discussed current procedures, recent advances, and challenges in HLA imputation methods, along with topics regarding reference panels. Since no one method outperforms the others in all aspects, it is important to understand the advantages of each method and use or integrate different methods according to the situation. In general, newer reference panels contain more information covering wider variations and higher resolution of HLA typing. Therefore, HLA imputation methods should evolve with more learning capacity and higher computational performance. We have expectations of the high learning capacity of deep neural networks as one of such methods. We also reviewed the findings obtained from fine-mapping in the MHC region and the hypothetical mechanisms of how MHC variants affect the susceptibility of autoimmune diseases. An effective approach in this field is to compare the different risks among HLA alleles and their biochemical functions validated by experimental techniques. Thus, in this sense, reliable HLA imputation methods and informative fine-mapping would be essential for further understanding of the immunopathology of autoimmune diseases.
